# Health-promoting lifestyle behaviors and their associated factors among pregnant women in Debre Markos, northwest Ethiopia: a cross-sectional study

**DOI:** 10.3389/fgwh.2024.1468725

**Published:** 2025-01-03

**Authors:** Getachew Tilaye Mihiret, Belsity Temesgen Meselu, Kumlachew Solomon Wondmu, Temesgen Getaneh, Nurilign Abebe Moges

**Affiliations:** ^1^ Department of Midwifery, College of Medicine and Health Sciences, Debre Markos University, Debre-Markos, Ethiopia; ^2^ Department of Public Health, Amhara Public Health Institute, Bahir Dar, Ethiopia

**Keywords:** antenatal care, factors, health-promoting lifestyle, pregnant women, Ethiopia

## Abstract

**Introduction:**

Promoting healthy lifestyle behaviors during pregnancy is a crucial health promotion strategy that could reduce pregnancy-related complications that may harm women and their fetuses. However, very few studies have assessed the prevalence of health-promoting lifestyle behaviors among pregnant women in Ethiopia. This study aimed to evaluate the extent and associated factors of health-promoting lifestyle behaviors among pregnant women in public health institutions in Debre Markos, northwest Ethiopia.

**Methods:**

An institutional-based cross-sectional study was conducted among 275 pregnant women who were recruited using a systematic random sampling technique from 19 April to 19 May 2021. A face-to-face interview-administered questionnaire was used to collect the data. The data were analyzed using SPSS version 25. Multivariable binary logistic regression was used to identify the factors associated with the outcome variable. adjusted odds ratio (AOR), with a 95% confidence interval (CI) were used to measure the strength of the associations at a *p*-value <0.05.

**Results:**

The average mean score for health-promoting lifestyle behaviors was 2.68 (±0.38). Factors such as rural residency (AOR = 0.29; 95% CI = 0.10–0.82), family size (≥5) (AOR = 0.25; 95% CI = 0.08–0.79), being the decision-maker for economic expenses (AOR = 0.34; 95% CI = 0.14–0.84), and average monthly income (AOR = 0.15; 95% CI = 0.04–0.59) were found to be significantly associated with health-promoting lifestyle behaviors during pregnancy.

**Conclusion:**

Approximately two-thirds of participants demonstrated better (healthier) health-promoting lifestyle behaviors during their pregnancy. To reduce unhealthy lifestyle-related maternal morbidity and mortality in Ethiopia, it is important to encourage health-promoting activities through health education and antenatal care follow-up with an emphasis on women who reside in rural areas, and who have a high family size, low income level, and have husbands who are the primary decision-makers for their economic expenses.

## Introduction

Health promotion is a crucial determinant and strategy for individuals to enhance and maintain their health. The World Health Organization (WHO) defines health promotion as “a process of enabling people to increase control over, and to improve their health” (1). Health-promoting lifestyle behaviors (HPLBs) are a key health promotion strategy aimed at improving or maintaining wellbeing or reducing the incidence of illness (2, 3).

Health-promoting lifestyle behaviors encompass individuals’ actions and perceptions on various levels of health promotion, including physical activity, nutritional management, spiritual growth, health responsibility, establishing good communication with others, and controlling and reducing stress in daily life (4–6).

According to Walker et al. (7), HPLBs have six dimensions: physical activity, nutrition, stress management, health responsibility, interpersonal relationships, and spiritual growth.

Health-promoting lifestyle behaviors are a key determinant of health and disease prevention for individuals, families, and society (8, 9), accounting for 60% of an individual's health and quality of life (10). In the general population, unhealthy lifestyle behaviors are the main reason for death across the globe (9); they account for 40%–50% and 70%–80% of deaths in developing and developed countries, respectively (11). Embracing proper HPLBs enables people to establish a pattern of self-initiated perceptions and actions that support self-care behaviors (12) and reduce the impact of non-communicable diseases (13).

Poor nutritional management remains a crucial factor in determining public health in Ethiopia (14). In addition, the country has seen a troubling increase in non-communicable diseases in recent times, which can be attributed to changes in lifestyle caused by economic development (15).

Even though HPLBs are a way of life and determine the health and wellbeing of all groups of populations (9, 10), pregnant women need special attention due to the physiological changes during pregnancy that significantly impact their health and their HPLBs (16). Neglecting to improve HPLBs during pregnancy can have serious short and long-term consequences for the growth and development of the fetus and for the long-term health of the mother (17). Each year, unhealthy behaviors and their sequelae account for 18 million deaths among reproductive-age women (18).

Improper HPLBs during pregnancy increase the likelihood of obesity, gestational diabetes, pregnancy-induced hypertension, antepartum hemorrhage, complications during labor and delivery, antenatal and postnatal depression, infection, hospital admission, and length of stay in neonatal intensive care units (9, 17, 19). Pregnancy-related complications and poor lifestyle behaviors related to stress management for existing complications can also lead to preterm birth, low birth weight, and pregnancy-induced hypertension. Furthermore, these behaviors also affect women's attachment and bonding with their newborns (19–22). Moreover, unhealthy behaviors such as smoking, drinking alcohol, and drug abuse increase the risk of intrauterine growth restriction, premature rupture of membranes, exposure to carcinogens, and early neonatal death (23).

According to different studies, overall HPLBs among pregnant women were found to be moderately prevalent (16, 19, 24, 25). However, the determinants of women's HPLBs vary in different societies based on their particular socio-cultural and economic contexts (6). During pregnancy, national health policies and the physical environments of countries are also significantly associated with HPLBs (20, 26).

The WHO recommends comprehensive healthcare that includes risk prevention and health promotion to ensure safe motherhood and improve the health of the fetus (27). Promoting healthy lifestyle behaviors during pregnancy is one of the key health promotion strategies to improve preventable pregnancy-related complications that could harm women and/or their fetus (9, 16).

Health promotion has been incorporated into healthcare services in Ethiopia with the aim of promoting healthy behaviors during pregnancy and childbirth and enhancing the wellbeing of both mothers and fetuses (28, 29). Assessing the existing prevalence of HPLBs and their contributing factors is considered crucial for encouraging HPLBs among pregnant women (16). However, there have been limited studies conducted on the prevalence of health-promoting lifestyle behaviors among pregnant women in Ethiopia. Previous studies in Ethiopia focused on specific aspects of the health-promoting lifestyle profile-II (HPLP-II) to assess the health-promoting behaviors of pregnant women. This study focused on determining the existing prevalence of HPLBs among pregnant women using a standardized tool that will help policymakers in developing effective strategies to address preventable lifestyle-related fetal and maternal morbidities and mortality, ultimately improving the quality of life in society. This study aimed to evaluate the prevalence of HPLBs and their associated factors among pregnant women attending antenatal care (ANC) units at public health institutions in Debre Markos, northwest Ethiopia.

## Methods

### Study design, area, and period

An institutional-based cross-sectional study was undertaken between 19 April to 19 May 2021 to assess the prevalence of HPLBs and their associated factors among pregnant women in public health institutions in Debre Markos, northwest Ethiopia. Debre Markos is located 300 km from Addis Ababa, the capital city of Ethiopia, and 265 km from Bahir Dar, the capital city of the Amhara regional state. The town has four governmental health centers and one specialized public hospital that provide preventive, curative, and rehabilitative health services, including ANC follow-ups.

### Source population

All pregnant women who visited the antenatal care units at the public health institutions in Debre Markos were considered the source population.

### Study population

The study population consists of pregnant women who attended the antenatal care units at the public health institutions in Debre Markos during the study period and met the eligibility criteria.

### Inclusion and exclusion criteria

All pregnant women who had visited the antenatal care units at least once and were available at the time of data collection were included in this study. Participants who were mentally and physically incapable were not considered study participants from the outset.

### Sample size determination

The sample size was computed using the following formula for a single population proportion:

n=[(Za/2)2×p(1−p)]/d2]

This was based on a study conducted in Mekele, Ethiopia, on health promotion practices among pregnant women in which the proportion was 79.9% (30), and thus, using the following assumptions: 95% level of confidence, 5% degree of precision, and a 10% non-response rate, the final required sample size was determined to be 275. This sample size was then allocated proportionally to each health institution.

### Sampling method and procedure

**Figure 1:**
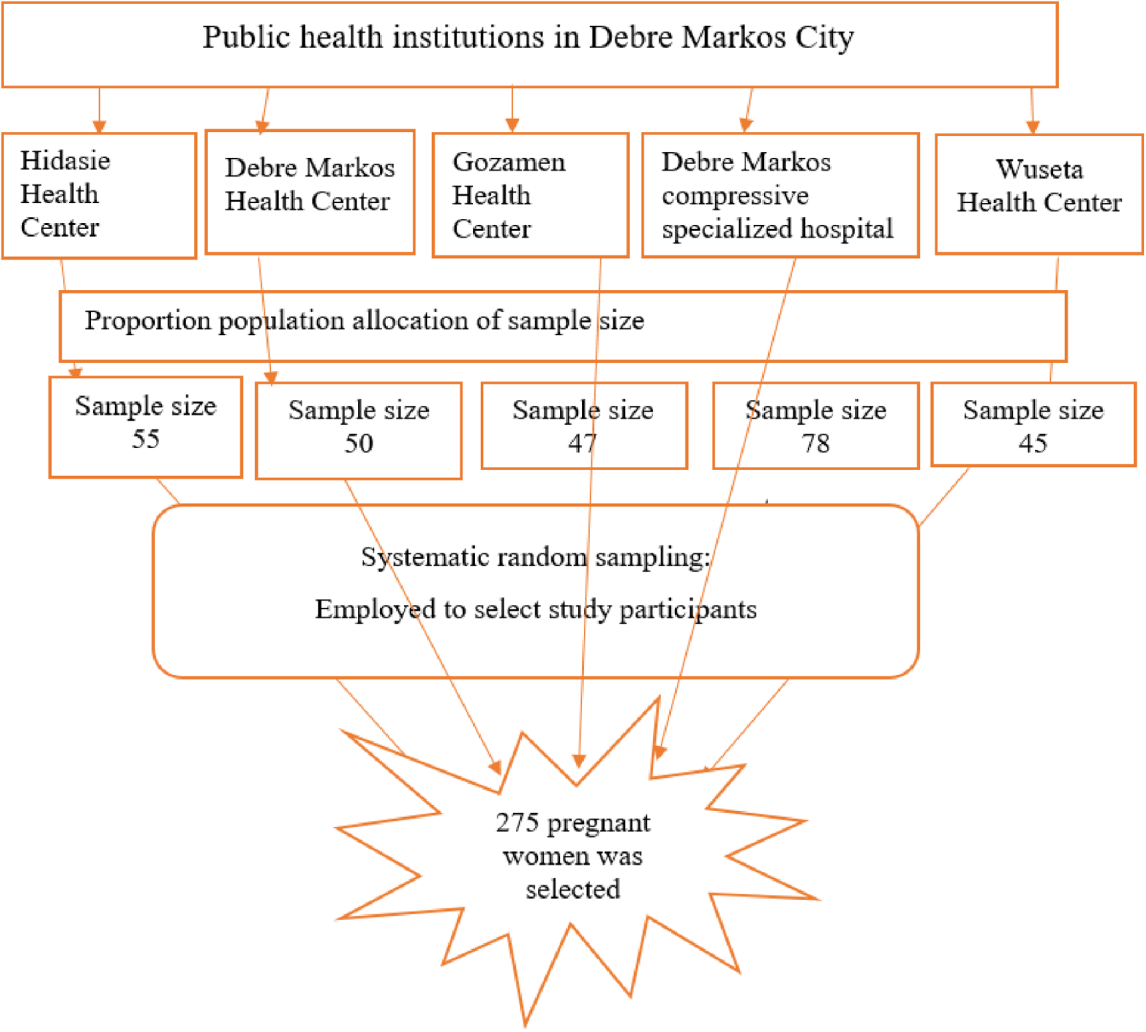
Schematic presentation of sampling procedure to select participants who attended antenatal care services at public health institutions in Debre Markos, northwest Ethiopia, 2021.

All the public health institutions in Debre Markos were included in this study. The ANC registration book from each health facility was used to proportionally distribute the calculated sample size and determine the sampling fraction (*k*) which was calculated using the population size divided by the sample size. According to the 6-month records from each public health institution, approximately 1,100 pregnant women were attending ANC services per month. The first study participant was selected using a simple random sampling technique among mothers who had an antenatal care follow-up on the day of data collection. A systematic random sampling technique was then employed until the required sample size was reached (Figure 1).

### Data collection tool and procedure

Five nurses with diplomas were enlisted for data collection and three nurses with BScs supervised the data collection process. The data were collected using an interviewer-administered questionnaire. The questionnaire included socio-demographic variables, obstetric characteristics, and HPLP-II. HPLP-II is a standardized tool developed by Walker et al. (4) based on Pender's health promotion model. It comprises 52 items on the six dimensions of HPLBs: health responsibility (9 items), physical activity (8 items), nutritional management (9 items), interpersonal relationships (9 items), spiritual growth (9 items), and stress management (8 items).

The questionnaire was initially prepared in English and then translated into Amharic (the local language) and back into English by two bilingual language experts to ensure consistency. Based on the experts’ feedback, two questions linked to “peak pulse rate and measuring pulse rate during physical exercise” were removed because most Ethiopian women are not familiar with measuring pulse rate. Three items related to nutritional behavior related to drinking at least 2 L of fluid per day, eating snacks between meals, and taking prescribed iron supplementation daily were added. The final version underwent face and content validity checks by five senior experts. A pilot test was then conducted with 30 participants who met the study criteria. The reliability of the final version of the HPLP-II tool was confirmed by computing Cronbach's alpha (*α*), which yielded a value of 0.8. This high reliability value indicates that the final tool produces reliable results.

### Study variables with operational definitions

Based on the mean score of the HPLP-II test items, overall HPLB was categorized into two levels (worse and better). Women with scores above or equal to the mean score of all the HPLP-II test items (≥2.50 on a scale ranging between 1 and 4) were considered to have better (healthier) HPLBs, and those with scores below the mean score were considered to have worse HPLBs (29, 31).

### Data quality control

To ensure the quality of the data, a 2-day training session was provided for both data collectors and supervisors by the principal investigator about the objective of the study, data collection tools, procedures, and how to fill out the questionnaire. Data collectors were supervised during the course of the data collection period. The overall process was then coordinated and controlled by the principal investigator. The completeness of the data was reviewed daily by the data collectors, supervisors, and principal investigators. Codes were given to the questionnaires during data collection.

### Data processing and analysis

The collected data were entered into Epi-data version 3.1 to minimize data entry errors and SPSS version 25 was used to analyze the data. A logistic regression model was employed to determine the possible predictors of healthier HPLBs in pregnant women. The model’s fitness was tested using the Hosmer–Lemeshow test (chi-square = 5.70, *p* = 0.68).

The variance inflation factors were analyzed to detect multicollinearity among the independent variables. Predictors with a *p*-value <0.25 in the bivariable logistic regression model were considered for the multivariable binary logistic regression model. Odds ratios with a 95% confidence interval were used to measure the strength of associations at a *p*-value <0.05.

### Ethical consideration

Ethical clearance was obtained from the Institutional Ethical Review Board of the College of Health Sciences, Debre Markos University (Approval No.: HSC/R/C/Ser/Co/316/11/13). Responsible officials and managers at each public health institution were contacted and permission was obtained. Written informed consent was obtained from each participant and they were informed that they had the right to withdraw from the study at any time.

## Results

### Socio-demographic characteristics

A total of 275 pregnant women were enrolled in the study. The mean age of respondents was 27.6 ± 4.7 years. In this study, 258 (93.8%) respondents were married. Of the study participants, 98.5% and 1.5% were from the Amhara and Oromo ethnic groups, respectively. Furthermore, 95.6% of the participants were Orthodox religious followers. This study also showed that 85.1% and 14.9% of the participants belonged to nuclear and extended families, respectively. The majority (88%) of the participants lived in urban residences (Table 1).

**Table 1:** Socio-demographic characteristics of pregnant women who attended antenatal care services at public health institutions in Debre Markos, northwest Ethiopia, 2021 (*n* = 275).

Variable	Category	Frequency	Percentage
Age (years)	15–24	73	26.5
	25–34	175	63.7
	≥35	27	9.8
Residence	Urban	242	88.0
	Rural	33	12.0
Marital status	Married	258	93.8
	Single/divorced/widow	17	6.2
Maternal education	No formal education	57	20.7
	Primary school	49	17.8
	Secondary school	72	26.2
	College and above	97	35.3
Maternal occupation	Government employee	75	27.3
	Private employee	19	6.9
	Merchant	58	21.1
	Housewife	111	40.4
	Other^a^	12	4.3
Husband education	No formal education	50	19.4
	Primary school	35	13.6
	Secondary school	58	22.5
	College and above	115	44.6
Husband occupation	Government Employee	96	37.2
	Private employee	40	15.5
	Merchant	77	29.9
	Farmer	21	8.1
	Other^b^	24	9.3
Average monthly income (in ETB)	<1,000	17	6.2
	1,000–2,000	57	20.7
	≥2,000	201	73.1
Family size	1–2	120	43.6
	3–4	126	45.8
	≥5	29	10.5
Decision-maker for expenses	Myself	34	12.4
	My husband	36	13.1
	Both	205	74.5

ETB, Ethiopian birr.

^a^ Daily laborer and student.

^b^ Driver and daily laborer.

### Obstetrical characteristics

In this study, unintended pregnancy was found among 11.6% of respondents. More than three-fifths (60.7%) of the participating mothers were multigravida. Approximately one-fifth (18.9%) of the participants had a history of miscarriage in a previous pregnancy and 128 (46.5%) respondents were in their third trimester (Table 2).

**Table 2:** Obstetrical characteristics of the pregnant women who attended antenatal care services at public health institutions in Debre Markos, northwest Ethiopia, 2021.

Variable	Category	Frequency (*n* = 275)	Percentage
Parity	0	127	46.2
	1–2	133	48.3
	≥3	15	5.5
Duration of pregnancy	First trimester	39	14.2
	Second trimester	108	39.3
	Third trimester	128	46.5
Antenatal care visit	First	61	22.2
	Second	58	21.1
	Third	79	28.7
	Fourth	77	28
Mode of delivery of last pregnancy	SVD	148	88.6
	C/S	19	11.4
Mid upper arm circumference (cm)	<23	75	27.3
	≥23	200	72.7
Alcohol during pregnancy	Yes	54	19.6
	No	221	80.4

SVD, spontaneous vaginal delivery; C/S, cesarean section; cm, centimeter.

### Descriptive statistics for HPLP-II

The mean score of HPLP-II was computed using all the test items in each subscale, each of which was scored between 1 and 4. The mean HPLP-II score of study participants was 2.68 (±0.38). The highest mean score was found in the spiritual growth section and the lowest mean score was obtained in the physical activity section of HPLP-II, with mean scores of 3.22 (±0.50) and 1.78 (±0.56) respectively (Table 3).

**Table 3:** Descriptive statistics for HPLP-II.

Subscales of HPLP-II	Mean (±SD)	Range (min–max)
Health responsibility	2.55 ± 0.59	1.33–4.00
Physical activity	1.78 ± 0.56	1.00–4.00
Nutrition management	2.75 ± 0.51	1.58–4.00
Spiritual growth	3.22 ± 0.50	1.89–4.00
Interpersonal relationships	3.00 ± 0.56	1.75–4.00
Stress management	2.42 ± 0.48	1.50–4.00
Overall HPLP-II	2.68 ± 0.38	1.85–3.57

HPLP-II, health-promoting lifestyle profile-II; SD, standard deviation.

### Health-promoting lifestyle behaviors of the respondents

**Figure 2:**
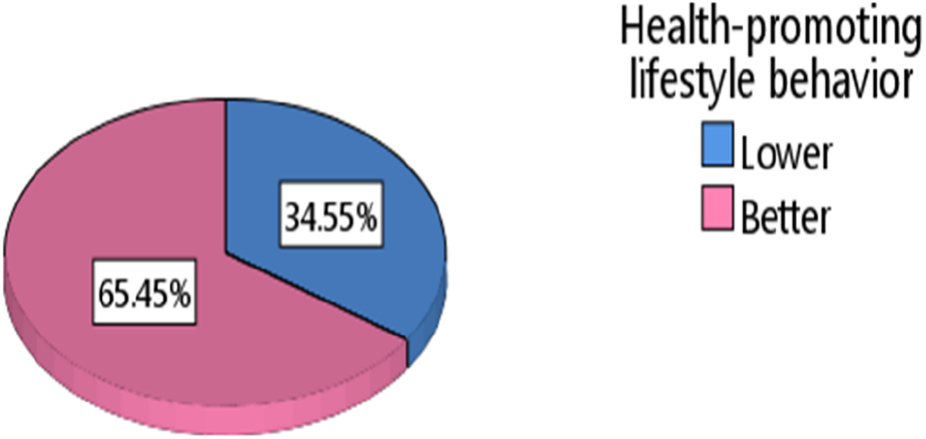
Prevalence of health-promoting lifestyle behaviors among pregnant women who attended antenatal care services at public health institutions in Debre Markos, northwest Ethiopia, 2021.

The overall prevalence of better (healthier) HPLBs among pregnant women who had ANC follow-ups at public health institutions in Debre Markos was 65.5% (95% Cl: 59.5–71.1) (Figure 2).

### Differences in mean score for HPLBs according to the independent variables

Based on the one-way analysis of variance (ANOVA), a statistically significant mean score difference was found for HPLBs in relation to women's educational levels (*p*-value: 0.00). The results of Tukey's *post-hoc* statistical test revealed that women's HPLBs improved as their educational level increased. The mean score for HPLBs was also significantly different among maternal occupational levels (*p*-value = 0.00); Tukey's *post-hoc* statistical test confirmed that housewives had a significantly lower mean score for HPLBs compared with government employees (*p* = 0.00).

Furthermore, the result of a one-way ANOVA shows a statistically significant difference in mean score for HPLBs due to husbands’ educational levels (*p* = 0.00); the result of Tukey's *post-hoc* statistical test indicated that a woman husband’s educational level had a statistically significant effect on her HPLBs. This means that a woman's HPLBs are significantly improved when their husband’s educational level is higher.

Moreover, based on the one-way ANOVA, the HPLBs of pregnant women significantly varied due to their average monthly income levels. Using Tukey's *post-hoc* statistical test, it was revealed that receiving more than or equal to 2,000 Ethiopian birrs (ETB) significantly improved the mean score for HPLBs compared to receiving less than 1,000 or between 1,000 and 2,000 ETB (*p* = 0.00).

Furthermore, the HPLBs of pregnant women significantly varied due to who was the primary decision-maker for family economic expenses (*p* = 0.00). According to Tukey's *post-hoc* statistical test, worse HPLBs were found among women whose husbands were the primary decision-makers for their economic expenses compared to the women and their husbands making decisions jointly (*p* = 0.00) (Table 4).

**Table 4:** Independent *t*-test and one-way ANOVA comparisons of the independent variables with the mean scores for health-promoting lifestyle behaviors (*n* = 275).

Variable	Category	Frequency	Mean for HPLB	Statistical test
Age (years)	15–24	73	2.69 (±0.33)	*F* = 3.30
	25–34	175	2.70 (±0.39)	*P* = 0.04
	≥35	27	2.50 (±0.38)	
Residence	Urban	242	2.71 (±0.36)	*t* = 4.42
	Rural	33	2.42 (±0.36)	*p* = 0.00
Maternal education	No formal education	57	2.48 (±0.35)	*F* = 16.06
	Primary school	49	2.55 (±0.34)	*P* = 0.00
	Secondary school	72	2.70 (±0.36)	
	College and above	97	2.84 (±0.34)	
Maternal occupation	Government employee	75	2.82 (±0.36)	*F* = 5.48
	Private employee	19	2.79 (±0.41)	*P* = 0.00
	Merchant	58	2.66 (±0.36)	
	House wife	111	2.58 (±0.36)	
	Other^a^	12	2.61 (±0.41)	
Husband education	No formal education	50	2.46 (±0.35)	*F* = 12.94
	Primary school	35	2.75 (±0.36)	*P* = 0.00
	Secondary school	58	2.61 (±0.37)	
	College and above	115	2.81 (±0.34)	
Average monthly income (in ETB)	<1,000	17	2.30 (±0.31)	*F* = 22.94
	1,000–2,000	57	2.50 (±0.34)	*P* = 0.00
	≥2,000	201	2.76 (±0.35)	
Decision-maker for expenses	Myself	34	2.60 (±0.38)	*F* = 8.46
	My husband	36	2.47 (±0.33)	*P* = 0.00
	Both	205	2.73 (±0.37)	
Parity	0	127	2.67 (±0.33)	*F* = 4.19
	1–2	133	2.72 (±0.41)	*P* = 0.02
	≥3	15	2.43 (±0.37)	

HPLB, health-promoting lifestyle behavior; SD, standard deviation.

^a^ Daily laborer and student.

### Factors associated with health-promoting lifestyle behaviors

Among all the covariates, age, place of residence, maternal education, maternal occupation, average monthly income, family size, primary decision-maker for economic expenses, intended pregnancy, and number of ANC visits were found to have an association with HPLBs in the bivariable logistic regression at a *p*-value <0.25. However, in multivariable binary logistic regression, only place of residence, family size, primary decision-maker for economic expenses, and average monthly income were identified as statistically significant factors for healthier HPLBs at a *p*-value <0.05. The Hosmer–Lemeshow test was performed (Chi-square = 5.70; *p* = 0.68), indicating that the model was a good fit with the observed values.

The findings of this study show that rural women were 71% less likely to have healthier HPLBs compared to urban women (AOR = 0.29; 95% CI: 0.10–0.82). Furthermore, having a family size of more than or equal to five persons was associated with worse HPLBs compared to having less than or equal to two people in the family. Women with a family of more than or equal to five persons were 75% less likely to have healthier HPLBs compared to those with less than or equal to two people in their family (AOR = 0.25; 95% CI = 0.08–0.79).

Average monthly income was associated with healthier HPLBs in pregnant women. The odds of healthier HPLB decreased by 0.15 times (95% CI: 0.04–0.59) for women with a monthly income of less than 1,000 Ethiopian birrs compared to those with an income of greater than or equal to 2,000 Ethiopian birrs. Being the primary decision-maker for family economic expenses was also associated with HPLBs in pregnant women. The odds of healthier HPLBs decreased by 0.34 times (95% CI: 0.14–0.84) for women who had husbands who were the primary decision-makers for their economic expenses compared to when both the women and their husbands made the decisions jointly (Table 5).

**Table 5:** Factors associated with health-promoting lifestyle behaviors among pregnant women in Debre Markos, northwest Ethiopia, 2021 (*n* = 275).

Variable	Category	HPLBs		Odds ratio (95% CI)	
		Better	Worse	COR	AOR
Age (years)	15–24	47	26	1	1
	25–34	123	52	1.31 (0.73–2.33)	1.40 (0.70–2.81)
	≥35	10	17	0.33 (0.13–0.84)	0.75 (0.23–2.47)
Residence	Urban	171	71	1	1
	Rural	9	24	0.16 (0.07–0.35)	0.29 (0.10–0.82)*
Maternal education	No formal education	25	32	0.19 (0.09–0.39)	0.70 (0.23–2.14)
	Primary school	25	24	0.25 (0.12–0.54)	0.42 (0.16–1.08)
	Secondary school	52	20	0.63 (0.31,1.30)	0.88 (0.35–2.22)
	College and above	78	19	1	1
Maternal occupation	Government employee	60	15	1	1
	Private employee	14	5	0.70 (0.22–2.25)	0.85 (0.23–3.14)
	Merchant	41	17	0.60 (0.27–1.34)	0.89 (0.31–2.57)
	Housewife	58	53	0.27 (0.14–0.54)	0.58 (0.23,1.49)
	Other^a^	7	5	0.35 (0.10–1.26)	0.75 (0.15–3.72)
Average monthly income (in ETB)	<1,000	4	13	0.11 (0.03–0.34)	0.15 (0.04–0.59)**
	1,000–2,000	27	30	0.31 (0.17–0.58)	0.62 (0.29–1.34)
	≥2,000	149	52	1	1
Family size	1–2	83	37	1	1
	3–4	87	39	0.99 (0.58–1.71)	1.08 (0.56–2.11)
	≥5	10	19	0.24 (0.10–0.55)	0.25 (0.08–0.79)*
Decision-maker for expenses	Myself	21	13	0.70 (0.33–1.49)	0.70 (0.25–1.93)
	My husband	16	20	0.35 (0.17–0.71)	0.34 (0.14–0.84)*
	Both	143	62	1	1
Antenatal care visit	First	42	19	1.49 (0.73–3.02)	1.82 (0.79–4.22)
	Second	42	16	1.77 (0.85–3.69)	1.77 (0.74–4.24)
	Third	50	29	1.16 (0.61–2.22)	1.23 (0.56–2.70)
	Fourth	46	31	1	1
Unintended pregnancy	Yes	164	79	1	1
	No	16	16	0.48 (0.23–1.01)	1.45 (0.54–3.88)

1 = Reference; COR: crude odds ratio; AOR: adjusted odds ratio; HPLBs, health-promoting lifestyle behaviors; Hosmer–Lemeshow test for adjusted model: chi-square = 5.70, *p* = 0.68.

^a^ Daily laborer and student.

*p < 0.05; **p < 0.01.

## Discussion

This study investigated the prevalence of HPLBs and their related factors among pregnant women who attended ANC follow-ups. Approximately two-thirds of participants demonstrated healthier health-promoting lifestyle behaviors during their pregnancy. This prevalence was inconsistent with a study conducted in Mekele, Ethiopia (79.9%) (30). This variation may be due to differences in assessment tools and study settings. The study in Mekele only used some components of HPLP-II and only included urban women, while this study used all the components of HPLP-II and included both rural and urban women. Thus, the variance might also be due to socio-cultural differences among the study populations.

According to this study, the mean score for HPLP-II was 2.68 (±0.38), which is comparable with other studies conducted among pregnant women in Iran (9), Taiwan (24), Jordan (32), and Turkey (25), and reproductive-age women in Iran (26). Overall, the HPLP-II scores of pregnant women were found to be above the average score of HPLP-II from these studies (16, 19, 24, 25), although it was still lower than expected. This implies that HPLBs among pregnant women are comparable across different countries, despite real variances in women's socio-demographic and economic statuses.

Among the six subscales of HPLB, the highest mean score was found in spiritual growth, with a mean score of 3.22 (±0.50), comparable to a study conducted in Turkey (25). In contrast, this finding is inconsistent with studies conducted in Iran (9) and Taiwan (24), which found the highest mean score in interpersonal relationships. Possible explanations for these differences might be the sampling technique and the duration of pregnancy of the respondents as the study in Iran used a convenience sampling technique and most of the respondents were in their second trimester, while our study used a systematic random sampling technique and most of the participants were in their third trimester.

This study found a lower mean score in the stress management and physical activity areas of HPLB. The physical activity lifestyle was the lowest relative to the other subscales of HPLB. This is consistent with other studies undertaken among pregnant women (16, 19, 24, 25) and reproductive-age women (26). This shows that women have lower physical activity lifestyles even though there are economic and cultural differences among reproductive-age women. This implies that a sedentary lifestyle is a challenge for reproductive-age women in most countries.

This study showed that participants who lived in rural areas had a lower chance of engaging in health-promoting activities than urban dwellers. A possible justification for this might be the fact that urban women may have easy access to health facilities, health education, and other social facilities. However, women who reside in rural areas have poor awareness of health-promoting activities. A study that was conducted in rural areas in Hungary showed that the economic development of the settlements was significantly associated with the HPLBs of pregnant women (33).

Women who had more than or equal to five family members in their house had worse HPLBs compared with those who had less than or equal to two family members in their house. This is in line with a study conducted in Afghanistan (6). A possible reason might be that large family size increases a woman's household responsibility, which leads to a reduction in their participation in health-promoting activities. In contrast, a study undertaken in Iran among pregnant women shows a substantial correlation between family size and levels of HPLBs. Women with more family members reported a high mean score for HPLBs. The sample size and socioeconomic differences between Ethiopian and Iranian women may be the possible causes of this discrepancy.

This study indicates that, as average monthly income increases, the HPLBs of pregnant women also increase. Studies conducted in Ethiopia (30), Iran (2, 6, 9), and Taiwan (24) also found similar results. Women who have more money are less likely to worry about where they get the money to engage in health-promoting activities, and this has a positive effect on their HPLBs (34). Women's HPLBs are also heavily influenced by their role as the primary decision-makers for family economic expenses. This was demonstrated by the fact that when husbands make decisions on their own in this study, the mean HPLB scores decreased (9). Thus, the women may miss out on participating in various health-promoting activities.

According to the results of the independent *t*-test and one-way ANOVA, higher educational and occupational levels and higher socioeconomic status have a positive effect on HPLBs. Similar evidence was found in studies in Turkey (19, 25). However, this study showed that older and multiparous women had a lower mean score for HPLP-II. This finding was not similar to the study findings in Turkey (19, 25). A possible reason for this discrepancy might be that in Ethiopia, older women and multiparous women may not get the opportunity to engage in different health-promoting activities due to cultural constraints and household responsibilities. Evidence shows that the determinants of HPLBs vary in different societies based on their particular socio-cultural and economic contexts (6).

## Limitation

Despite filling a gap in the literature, this study has its own limitations. First, due to the cross-sectional nature of the study, cause-and-effect relationships were not determined. Second, since this study only included pregnant women who had made ANC visits at public health institutions, it may not be generalizable to the whole population of pregnant women.

## Conclusion

This study revealed that approximately two-thirds of the respondents had healthier HPLBs during their pregnancy. Factors such as rural residency, family size, being the decision-maker for economic expenses, and average monthly income were found to be significantly associated with health-promoting lifestyle behaviors during pregnancy. To reduce unhealthy lifestyle-related maternal morbidity and mortality in Ethiopia, it is important to encourage health-promoting activities during health education and antenatal care follow-ups with an emphasis on women who reside in rural areas, and who have big families, a low income level, and husbands who are the primary decision-makers for their economic expenses.

## Data Availability

The datasets presented in this study can be found in online repositories. The names of the repository/repositories and accession number(s) can be found here: 10.5281/zenodo.7336223.
